# Polyetherimide-Reinforced Smart Inlays for Bondline Surveillance in Composites

**DOI:** 10.3390/polym14183816

**Published:** 2022-09-13

**Authors:** Chresten von der Heide, Julian Steinmetz, Oliver Völkerink, Patrick Makiela, Christian Hühne, Michael Sinapius, Andreas Dietzel

**Affiliations:** 1 Institute of Microtechnology, Technische Universität Braunschweig, 38124 Braunschweig, Germany; 2 Institute of Mechanics and Adaptronics, Technische Universität Braunschweig, 38106 Braunschweig, Germany; 3 Institute of Composite Structures and Adaptive Systems, German Aerospace Center (DLR), 38108 Braunschweig, Germany

**Keywords:** thin-film sensors, foil sensors, composite structures, structural bonding, multifunctional bondline, function conformity, sensor integration, structural health monitoring

## Abstract

An integrable sensor inlay for monitoring crack initiation and growth inside bondlines of structural carbon fiber-reinforced plastic (CFRP) components is presented. The sensing structures are sandwiched between crack-stopping poly(vinyliden fluoride) (PVDF) and a thin reinforcing polyetherimide (PEI) layer. Good adhesion at all interfaces of the sensor system and to the CFRP material is crucial, as weak bonds can counteract the desired crack-stopping functionality. At the same time, the chosen reinforcing layer must withstand high strains, safely support the metallic measuring grids, and possess outstanding fatigue strength. We show that this robust sensor system, which measures the strain at two successive fronts inside the bondline, allows to recognize cracks in the proximity of the inlay regardless of the mechanical loads. Feasibility is demonstrated by static load tests as well as cyclic long-term fatigue testing for up to 1,000,000 cycles. In addition to pure crack detection, crack distance estimation based on sensor signals is illustrated. The inlay integration process is developed with respect to industrial applicability. Thus, implementation of the proposed system will allow the potential of lightweight CFRP constructions to be better exploited by expanding the possibilities of structural adhesive bonding.

## 1. Introduction

Adhesive bonding is ideally suited to join lightweight components made from composite materials because the load is transferred with only low stress peaks in the adherends. In contrast to bolted joints, load bearing fibers are not cut, and thus the composite material is not weakened. In addition, weight savings of up to 15% as well as fabrication cost savings of up to 30% through reductions in both procurement and life-cycle maintenance can be achieved by full implementation of adhesive bonding [1,2,3,4]. Despite the clear advantages, adhesively bonded joints have so far been used almost exclusively for non-load-critical structures, as reliability is still a major concern, especially for structural bonding in aviation [5]. Various possible bondline defects such as disbonds, voids, cracks, foreign material inclusions, porosities, poor cure, and weak bonds, as well as sensitivity to environmental or physico-chemical conditions make it challenging to ensure a certain level of adhesive strength [2,6]. Thus, critical primary bonded joints are still accompanied by additional *fail-safe* mechanical fasteners (sometimes referred to as *chicken-rivets*), which diminish the benefits of adhesive bonding [7,8,9]. Regulation authorities make clear requirements for certification of adhesively bonded joints whose failure would mean a catastrophic loss to the overall structure [10]. While proof testing of each bond is costly and inefficient, reliable non-destructive inspection techniques do not exist yet [6]. Instead of proof testing, the regulations can also be fulfilled by limiting the possible disbond size accompanied by some kind of self-triggered repair request. For this, various adhesive layer monitoring systems with different sensory detection principles have been described in the literature [11,12,13,14]. In addition, upon a partial disbond, sensor-equipped design features have to ensure that a critical size of intact bond area is maintained under all circumstances [15]. Thus, the toughening of the adhesive bondline is a crucial part of the superordinate system, for which various techniques exist [16,17].

By embedding a strip of a ductile polymer like poly(vinyliden fluoride) (PVDF) into the prepreg of the load-inducing adherend prior to curing, surface toughening (ST) by disbond-stopping features (DSFs) can be realized in a simple way that is compatible with industrial fabrication [18,19]. To expand this concept with sensing capabilities, an easy to integrate smart inlay that combines crack sensing and stopping capability by forming a multifunctional disbond arrest feature (MDAF) was recently developed [20]. Strain sensor structures were applied directly onto the thermoplastic fluoropolymer. Although measurement data showed promising results and demonstrated bondline surveillance ability, electrical failures occurred quickly during fatigue testing. Load peaks at the filigree structures open to the adhesive layer were found to be a major source of defects. Encapsulation of the sensor structures using a second PVDF cover layer can be ruled out, since both layers would melt simultaneously during the carbon fiber-reinforced plastic (CFRP) integration process. Without mechanical reinforcement, the thin metallic micro structures could flow in the surrounding molten mass, leaving them distorted and destroyed after cooling.

Polyetherimide (PEI) material has a higher melting point than PVDF. Hence, it should remain stable during CFRP integration when the PVDF layer is completely melted, thereby preserving the original shape of the (sensor) structures. In addition, PEI possesses a higher Young’s modulus and exhibits excellent adhesion to the CFRP matrix, as shown before [21]. By introducing an additional polymer layer of PEI on which sensor structures are placed, durability in fatigue testing of inlay-equipped adhesive joints shall be improved, to achieve function-compliant behaviour (adhesive load transfer, crack stop, and crack detection). The lithographic structures on the PEI substrate shall be encapsulated by the crack-stopping PVDF layer, which provides improved handling robustness and increases their distance to the stress peaks at the PVDF surface.

In this article, the enhanced inlays are investigated with regard to functional compliance. At best, the sensory inlay should stay intact and supersede the fatigue test loading cycles with the crack being arrested in the proximity of the first DSF. To assess the improved inlay design and assure a beneficial effect of the PEI layer introduction, finite element (FE) simulations of the strain fields in cracked lap shear (CLS) specimens were conducted. Then, corresponding specimens were built and tested. In the following, the experimental data is used to derive crack detection methods based on strain sensing.

## 2. Materials and Methods

### 2.1. Simulation

Abaqus/Explicit Version 2021 was used to solve the nonlinear 3D models. In order to reduce simulation time, the load was applied in a time period of 0.01 s, which is quicker than in the conducted experiments. The influence of shortening the time period was found to be negligible. The adherends made from composite material were modelled using a layer-wise approach with reduced integrated eight node linear solid elements (C3D8R). In the z-direction (the direction through the thickness of the sample) one element per layer was used. The element edge length in the y-direction (the direction of the shorter specimen side) was 1.0 mm for all elements. In the region of interest, the element edge length in x-direction (the direction of the longer specimen side) was set to 0.25 mm. In other regions, a coarser mesh with 1.0 mm was used to save computation time. The same was applied to the adhesive layer. The crack-stopping PVDF layer, however, was discretized with nine elements in the z-direction to get a strain gradient in the thickness direction. Another measure to save computation time was to build up a half model using symmetry in the xz-plane, assuming that the resulting error for 45°-plies had only a negligible effect. The material data from Marlett and Tomblin [22] were used to model the composite adherends made from HexPly 8552-IM7 in combination with a linear-elastic transversally isotropic material model. The film adhesive was modelled using the Drucker–Prager exponent model in combination with material parameters derived in previous work [23] to account for hydrostatic pressure-sensitive yielding. The hardening curve was taken from Tomblin et al. [24]. The PVDF material was modelled using von Mises plasticity and material data provided by Campus Plastics [25] from a similar PVDF material, Arkema Kynar 740.

### 2.2. Smart Inlay Fabrication and Integration

**Figure 1:**
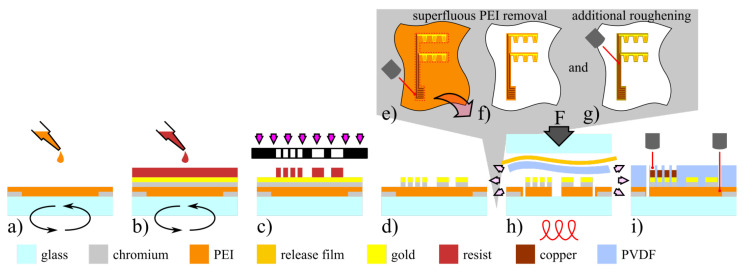
Smart inlay fabrication: (**a**) PEI spin coating; (**b**,**c**) metallic layer sputtering and lithography; (**d**) chemical wet etching; (**e**) PEI cutting; (**f**) superfluous PEI foil peel off; (**g**) roughening by means of fs-laser ablation; (**h**) PVDF encapsulation; (**i**) geometry cut and pad opening.

The inlay fabrication depicted in Figure 1 was based on a process described earlier [20]. A major change, however, is that the bottom substrate layer was produced by spin coating of a liquid 10 wt% PEI precursor based on polymer pellets diluted in trichlorethanol [26] at a spin speed of 1000 rpm on a 4 inch glass wafer. This was followed by hot plate curing for 2 min at 150 °C. After cooling, a second polymer layer was applied in the same manner before final curing was conducted at 220 °C for 10 min (see Figure 1a).

In order to promote adhesion to the PVDF-interface upon encapsulation, the PEI surface was modified by means of a laser workstation (microSTRUCT C, 3DMicromac) with a pulsed laser source (212 fs pulse length) emitting at a primary wavelength of 1030 nm in linear horizontal polarization. To rule out sudden crack propagation through the PEI/PVDF interface, the bottom PEI layer was cut and partially removed, leaving behind only the contoured regions supporting the sensor structures (see grey insert in Figure 1). This way, the PVDF DSF remained in direct CFRP contact after integration. In addition, the remaining PEI surface was roughened using less laser power. An isotropic pattern created by four scan lines rotated by 30° respectively, was used as a filling to create uniform abrasion. As the ablation threshold values for the metallic structures exceed those of the substrate polymer, they stayed unharmed while only the surrounding polymer is affected.

After sensor structuring and electroplating the PEI substrate was encapsulated (see Figure 1e) with a 100 μm thick PVDF foil using a similar process as for PVDF glass wafer fixation before [20]. In the vacuumized bonder (AB-1PV, Electronic Vision Co., Schärding, Austria), the PVDF foil was completely melted at 190°C while curing for 3 h at 1.5 bar.

Lastly, the outer smart inlay geometry was laser cut and peeled off the glass carrier wafer using tweezers. Integration into CFRP followed the *co-curing process* [20]. After adhesive bonding of both adherends, the composite plates were separated into the previously described CLS specimen geometry by saw cuts and equipped with a soldered plug to connect the sensors. The adherends are referred to as *lap* for the overlapping upper part and *strap* for the continuous bottom part.

### 2.3. Mechanical Testing

Various mechanical tests were conducted to investigate the sensory characteristics through static and dynamic testing of the inlay-equipped specimens.

#### 2.3.1. Inlay Calibration

**Figure 2:**
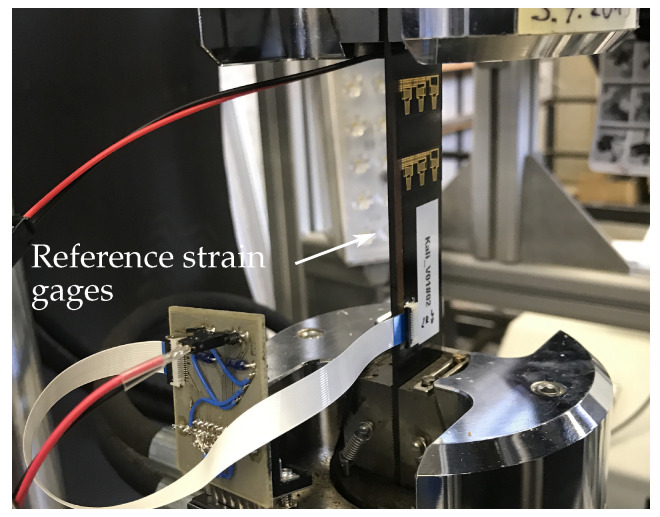
Calibration setup inside tensile rig with clamped open (lap-free) specimen.

To convert the electrical sensor signals into corresponding strain values, the inlay was first calibrated using a specimen with a constant cross-section (25 × 155 mm). Without an overlapping adherend, the strain was uniformly distributed and directly measured through the tensile rig (see Figure 2). For calibration, the specimen was loaded five times to an elongation of 1000 μm $m^{-1}$, ramping up and down within 10 s each. This was preceded by three identical cycles with subsequent zeroing of the displacement in order to eliminate slip, slack, and other falsifying influencing factors. In addition, two commercial quarter bridge strain gages were placed orthogonal to each other on the specimen backside. They serve as reference and for determination of the Poisson ratio of the layered composite structure.

#### 2.3.2. Quasi-Static Testing with Various Crack Lengths

CLS specimens with well-defined crack lengths and a straight crack front shape were fabricated by inserting square release films of different lengths during the adhesive bonding process. Thereby artificial crack lengths of 10, 16, and 23 mm were produced. Each specimen was subjected multiple times to an upramping tensile load of 5.104 kN (mean value of the cyclic load at 3000 μm $m^{-1}$ used during fatigue testing). Sensor signals were measured using a multi-channel strain gage amplifier (QuantumX MX1616B, HBM, Darmstadt, Germany).

#### 2.3.3. Dynamic Fatigue Testing

**Figure 3:**
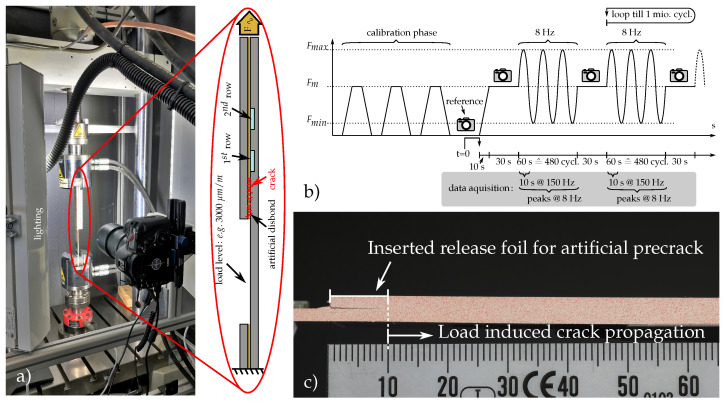
Dynamic fatique testing overview: (**a**) tensile test bench with enlarged CLS specimen sketch; (**b**) program overview of dynamic loading; (**c**) sideview picture after calibration run.

Fatigue testing of MDAF-equipped CLS specimens was conducted in a tensile rig (Zwick-Roell, Amsler HC25) (see Figure 3a). Forces were selected according to Table 1 such that the adhesive layer was overloaded, to force a slowly progressing crack growth. The lowest strain level corresponds to the maximum *limit load* for composite structures in aeronautical applications, which is the maximum design load that may occur during service life [27]. Moreover, typical ultimate strains in composites are 4000 μm $m^{-1}$ [1]. The selected sinusoidal loading maxima of 9.28 kN and 12.43 kN induce limit and ultimate strain in the slender bottom strap, respectively. Crack length was monitored by a large sensor camera (Canon EOS 5D Mark IV, Zeiss Milvus 2/100 M macro objective) with the external trigger fixed at one side of the test bench together with powerful LED lighting. Inlay sensors were again connected to the strain gage amplifier.

For crack length measurement, a threshold algorithm was applied to the images in Python. After cropping the images to remove the scale, the “skimage threshold_isodata” filter was used to delete the red speckles and obtain black and white images of the CLS specimen. The crack end was then visible as the black point furthest to the right. The crack origin was set manually in the first image of the measurement, so that the crack length is found by taking the difference of the corresponding x-coordinates in pixels. The length was converted to mm by a pixel-to-mm ratio obtained from the ruler in the image before cropping. It should be pointed out that the quantitative crack length in the CLS specimens is ambiguous. The rather thin crack opening in combination with the threshold algorithm led to a constant underestimation of the crack length. For that reason, the crack length estimate given by the algorithm was corrected by 5 mm based on a manual re-inspection and taking into account that the initial crack length of 10 mm due to the artificial disbond was already known. The correction did not alter the qualitative change in crack length determined by the algorithm.

Figure 3b exemplifies the cyclic loading process that ended after 1,008,000 cycles [4]. If crack propagation is successfully maintained inside the first DSF after test completion, operational fatigue strength can be concluded. After clamping, the respective specimen was loaded three times to the mean load level in order to eliminate possible mechanical displacements inside the rig or clamping, to open up the artificial precrack, and to synchronize the measurement devices. Below the clamped specimen, the internal load cell (Huppert, 1010-BPS-25kN-5/8′′) was used to zero the displacement value in the load-free status before a reference picture was taken. The testing then began by ramping up to the mean load level, where the first picture under load was taken. This was followed by the oscillation cycle, during which the specimen was subjected to a sinusoidal load at a frequency of 8Hz for 1 min. After these 480 cycles, the oscillation was stopped while the mean load level was maintained to open up the crack created. In the steady state, a high quality picture such as in Figure 3c was taken, where the crack stands out in form of a thin black line from the white painted sidewall of the specimen. An additional randomly distributed red *speckle pattern* was added by air brushing to later allow further investigation by means of particle tracing based on digital image correlation (DIC). Due to the long testing duration of about two days, efficient data acquisition was required to avoid large files. Therefore, only a 10 s snippet at high sampling rate was stored at the start of every 60 s oscillation phase. In data post-processing, these snippets were evaluated for mean and maximum strain values.

## 3. Smart Inlay Concept and Evaluation of Reinforced Design

### 3.1. Basic Inlay Functionality

**Figure 4:**
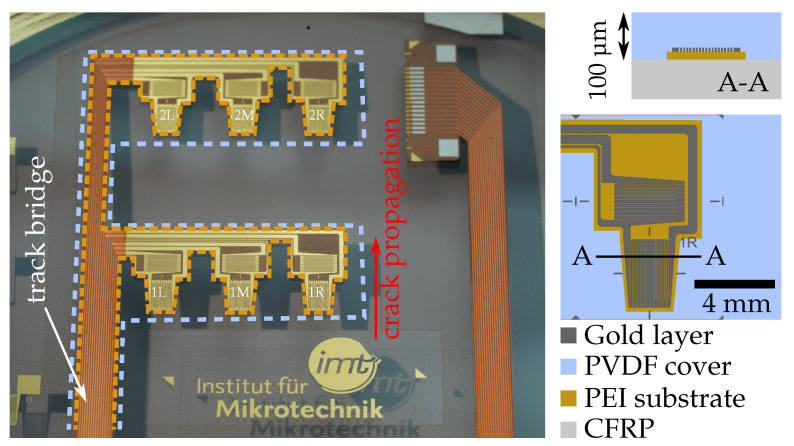
Smart Inlay (still on carrier wafer) with PEI-reinforced two-strip arrangement of sensors. To emphasize the shape of both polymer layers they are surrounded by an orange dotted line (for PEI) and a white dotted contour (for PVDF). Crack propagation direction towards the inlay is indicated in red. Sensor positions in row 1 and 2 are additionally marked L (left), M (middle), and R (right). The labeled track bridge forwards all electrical signals. Right: Schematics are showing the geometry of the sensors in cross-sectional and top view.

The inlay design (Figure 4) features six sensor nodes in a double strip design (three sensors each). The three sensors close to the emerging crack front in row 1 monitor its propagation, but may eventually fail upon arrival; meanwhile, the sensors in the second strip further behind shall remain functional to give a measure for the load on the structure as well as to detect unexpected crack continuation. The sensor connecting tracks on the left side are electroplated with copper to a thickness of about 8 μm to lower electrical resistance and improve their mechanical robustness.

Data exemplifying the stress peak and relief profile inside the adhesive layer starting at the overlap of a stained specimen has previously been provided [28]. Thus, as the crack advances through the bondline as depicted in Figure 4, the approximately 10 mm-wide stress profile shifts likewise. This means, the bondline stress profile inside an uncracked specimen decreases within 10 mm to a purely load-dependent value. In order to ensure that only real crack initiation rather then local stress peaks are detected, the first sensory strip is placed 15 mm away from the targeted crack start (artificial disbond length of 10 mm must be added). In the healthy bondline state, the same load-dependent sensor value will be measured by a second sensory strip with more clearance to the overlap edge. Thus, any sensor signal difference between both rows can be attributed directly to crack initiation.

**Figure 5:**
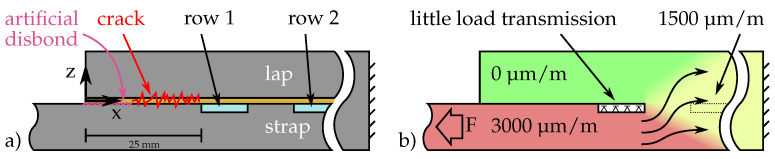
Simplified mechanical model of sensor zone: (**a**) specimen sideview with exaggerated crack depiction and inlay colored in light blue and (**b**) force transition flow into the overlapping adherend, indicated by arrows. The red (full load) to green (no load) color transition indicates the approximate stress. Little load is transferred at the polymer strip interface due to the low PVDF stiffness.

Figure 5 shows cross-sectional schematics illustrating a situation where the crack has reached the DSF such that load is transferred solely in the overlapping region behind it. As the overlapped section of the specimen is thicker, the force flow fans out into both adherends with increasing overlap length. Behind a certain transition region, strain is divided according to the ratio of the adherend thicknesses. For the used samples the overlapping region was twice as thick; thus, the strain is halved in its middle, which is in the adhesive layer. This means that once the crack has reached the inlay, the strain sensors in the first row will measure approximately the same value as if the DSF was not adhesively bonded to the overlapping CFRP part.

### 3.2. Strain Field Simulations

FE analyses were carried out to study the above-mentioned strain fields in the proximity of the crack-stopping PVDF layer inside the CLS specimen with variation of the crack lengths under static loading. These were evaluated in order to identify positions that are sensitive to crack growth and at the same time show strains which the sensor structures can resist.

In the model, a velocity loading of 100 mm $s^{-1}$ was applied on the strap-only side with a smooth amplitude to prevent oscillations in the model. All simulations were performed at a reaction force of 9.28 kN. The strap/lap doubled-up side of the modelled specimen was fully clamped. The adhesive was connected to the adherends and to the crack-stopping PVDF strips using tied constraints. Different from that, the PVDF inlays were attached to the adherends via merged nodes. The strain values presented and discussed in the following were evaluated at the element centroids by an Abaqus Python script using predefined element sets. It must be noted that alternating strain values occurred in the PVDF element row adjacent to the bondline. This is we attributed by the authors to strain localisation effects. To avoid this problem, the strain values were evaluated in the row below the interfacing elements.

In the beginning, the simulation was validated by values obtained with strain gages that were applied to the strap, and yielded a strain of 3000 μm $m^{-1}$ at the predefined load of 9.28 kN. Under equal loading, the FE-model showed 2900 μm $m^{-1}$, which is considered a sufficient match.

**Figure 6:**
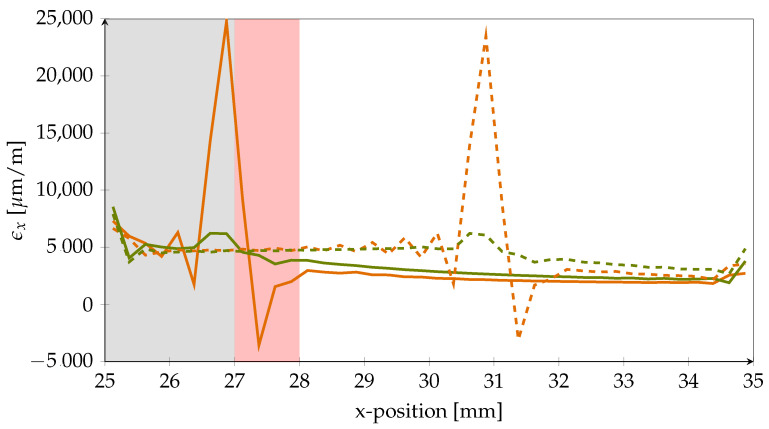
Nominal strain in the x-direction in PVDF at different positions. The orange lines correspond to the upper PVDF–adhesive-interface. Green lines represent the lower PVDF–CFRP-interface. Results for the two different crack lengths can be distinguished by the line type (solid = 27 mm, dashed = 31 mm). The grey background marks the simulated artificial disbond, while the red area is the resulting destructive zone for sensor structures because of high strain gradients.

First, the nominal strain in the x-direction ($\epsilon_{x}$) in the PVDF strip was evaluated for two different crack lengths at two different height levels which represent extreme positions; see Figure 6. On the one hand, the strains were investigated at the PVDF–adhesive-interface at the top of the PVDF strip (orange lines). On the other hand, the strains were evaluated at the bottom of the PVDF strip, which is the interface between the PVDF and the CFRP adherend (green lines). The solid lines in Figure 6 show the strains for 27 mm crack length, which means that the crack has intruded the first crack-stopping area by 2 mm. The dashed lines represent a crack length of 31 mm, which is equal to a crack intrusion of 6 mm.

With 24,800 μm $m^{-1}$, the highest strain was measured at the PVDF-adhesive-interface where the crack intruded the first PVDF strip. At the same position, the strain was with 6200 $\mu m\;m^{-1}$ much lower at the PVDF-CFRP-interface. However, the influence of the crack is still noticeable. The same held true for the second PVDF strip, where the crack was extended to 31 mm. This leads to the conclusion that positioning of the sensor structures close to the CFRP-interface beneath a covering layer is desirable, since the material stressing effort of the sensor strongly reduces with increasing distance to the adhesive-interface. Thus, sensor robustness is improved by lowering the stress peaks acting upon it if the crack intrudes the first stopping feature. In addition, the simulation reveals that the sensor measuring grid should be positioned with sufficient spacing to the front edge of the inlay to avoid the high strain gradients inside the approximately 1 mm-wide region behind the crack front, denoted as the *destructive zone*. Behind this zone, $\epsilon_{x}$ settles at a stable, well measurable value.

**Figure 7:**
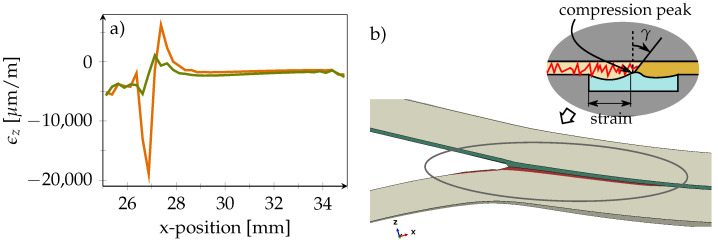
(**a**) 27 mm crack length. Nominal strain in z-direction in PVDF at different positions. Orange line represents PVDF-adhesive-interface while green line visualizes the PVDF-CFRP-interface. (**b**) 20 % exaggerated FE-deformation of strap. Insert shows sketch of PVDF strip deformation with intruded crack. Shear angle γ within adhesive layer is indicated.

Moreover, from the evaluation of strains in the x-direction, it can be seen that the PVDF material is elongated behind the progressed crack front (left side in the figure) and compressed in its current vicinity. This finding is supported by Figure 7a, which shows the strain $\epsilon_{z}$ in through-thickness direction. A simplified depiction of the PVDF strip deformation is shown in Figure 7b.

**Figure 8:**
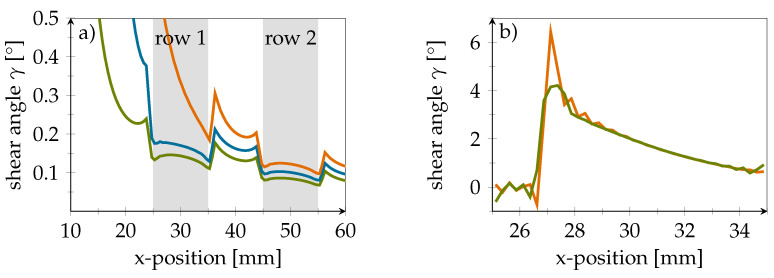
(**a**) Shear angle plot for different crack length. Green: 10 mm, Blue: 16 mm, Orange: 23 mm. Grey areas mark positions of PVDF inlays. (**b**) Difference in shear angle in xz-direction at the top and bottom of the PVDF inlay and a crack length of 27 mm. Orange line represents PVDF-adhesive-interface while green line visualizes the PVDF-CFRP-interface.

Figure 8a shows the course of the xz-shear angle γ within the adhesive layer at different crack lengths before the crack reaches the DSF. It can be seen that the shear angle γ in the inlay proximity is reduced. In Figure 8b, however, the crack has propagated into the inlay. Here, it can be seen that the nominal strain in the xz-direction, and thus the shear deformation, is reduced when moving away from the adhesive-interface.

At the PVDF–PVDF-interface, the shear strain is only 65% of the value at the PVDF–adhesive-interface. However, likewise to the observations for strains in the x-directions, the crack clearly shows in the strain curves at both positions. This indicates that the sensor should not be positioned directly at the adhesive-interface, although a crack in the adhesive is to be detected.

In preliminary testing of the inlays, a practical problem was caused by ripped off copper tracks in the track bridge area connecting the sensors with the solderable plug. As the tracks were in contact with the adhesive layer, high strains were induced and the crack propagated slowly, causing loss of sensor signals.

**Figure 9:**
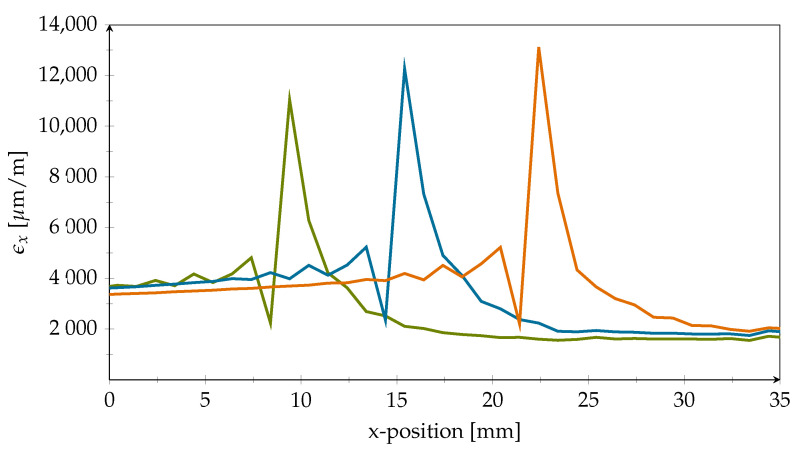
Nominal strain in the x-direction in track bridge for different crack lengths: green, 10 mm; blue, 16 mm; orange, 23 mm.

To investigate this issue further, the same model as above with an added strip of PVDF on the specimens side was used to evaluate the strains in the x-direction at three different positions of the track bridge. As track ripping was observed in the immediate transition area of the artificial disbond at *x* = 10 mm, the simulations were conducted for crack lengths of 10 mm, 16 mm, and 23 mm; see Figure 9.

From the plot, it can be seen that for a 10 mm crack length the maximum strain in the x-direction is higher than 11,000 μm $m^{-1}$. In addition, it can be seen that the maximum strain increases even further up to 13,000 μm $m^{-1}$ with increasing crack length. These high stresses explain the ripped-off tracks found in some experiments. Very similar results were obtained regardless of whether the inlay top at the adhesive layer-interface or the CFRP transition zone at the inlay bottom was evaluated. Since strains of this magnitude far exceed the robustness of metallic materials under continuous fatigue loading, milling of the lap immediately above the track bridge prevented damaging stress peaks during our experiments. Due to the elastic PVDF cover on top, the load transfer from the strap into the lap was very limited in this area. This adaptation enabled long term measurements. The wiring and signal transmission of smart inlay sensors, which in the future could potentially be integrated during industrial production of CFRP components, must be certain to take these findings into account.

The simulation results for strain in the x-direction confirmed the expected benefits of placing the sensor under a protective layer. As the ductile PVDF DSF deforms rather strongly at the adhesive layer-interface due to the sudden changes in material stiffness, the elastic material is incapable of providing the required support for the fragile measuring grids. The same analysis revealed a destructive zone of about 1 mm in width at the front edge of the DSF where stress gradients are steep (refer to Figure 6 for details). Due to the intentional overloading of the adhesive layer during fatigue testing, the crack emerges and propagates, but shall eventually stop in front of the DSF at 25 mm. This means that the resulting stress peak will stay in this position during most of the fatigue cycle, causing the depicted elevated stress profile in its proximity. Therefore, sensor structures on the inlay should be placed with a clearance of at least 1 mm to the inlay edge. Lastly, the PVDF inlay is heavily deformed in both the x- and z-direction. Peel load magnitude is quiet comparable to in-plane stresses; thus, adhesion of the sensor structures to the substrate must be strong.

## 4. Results

### 4.1. Smart Inlay Calibration

**Figure 10:**
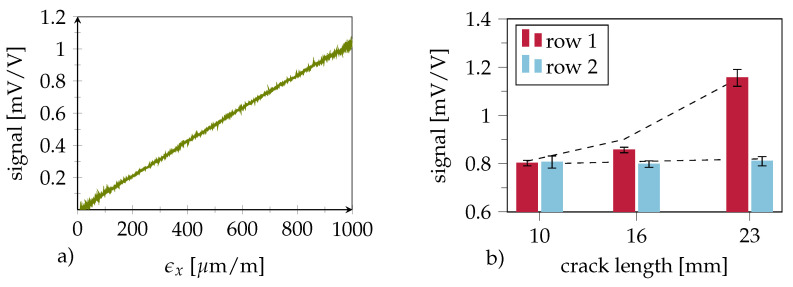
(**a**) Sensor calibration: Specimen was loaded using a ramp signal up to a maximum strain of 1000 μm $m^{-1}$. (**b**) Mean signal amplitudes for statically strained specimens with various artificial crack lengths. Standard deviation is represented in the form of brackets. FE simulated results are shown by a dashed line.

Smart inlay calibration through tensile loading (Figure 10a) showed a linear behaviour with respect to the longitudinal strain $\epsilon_{x}$. A peak signal amplitude of 1.02 mV$V^{-1}$ (the ratio of the measured bridge voltage $V_{diff}$ and supply voltage $V_{cc}$ at $\epsilon_{x}$ = 1000 μm $m^{-1}$) was measured. Considering the sensors’ half-bridge structure with orthogonal measurement grids and a Poisson ratio of ν=0.36 (derived for the specific CFRP layup; refer to Section 2.3 for details), Equation (1) [29] yields a gage factor of k=3.0:

(1) $$\frac{V_{diff}}{V_{cc}}=\frac{1}{4}\;\cdot \;k\;\cdot \;\epsilon_{x}\;\cdot \;\left(1+\nu \right)$$

As the measuring grids were fabricated from a thin layer of gold, this value seems rather high; however, it can be explained by the underlying chromium layer, which slightly alters the electro-mechanical properties.

### 4.2. Crack Sensing in Quasi-Static Testing

Over the course of their life cycle, structural bonds must endure varying load conditions. A single strain-sensitive sensor is incapable of distinguishing between load-induced strains and those caused by crack initiation. The smart inlay concept is based on recognizing strain gradients between two consecutive measurement locations at different distances to the crack front. Here, load-induced signals in the healthy crack-free adhesive layer are identical at both positions due to the uniform load distribution inside the bondline. In the case of a crack, however, the stress signals differ as a function of the distance from the crack front due to the decreasing load transfer into the lap.

**Figure 11:**
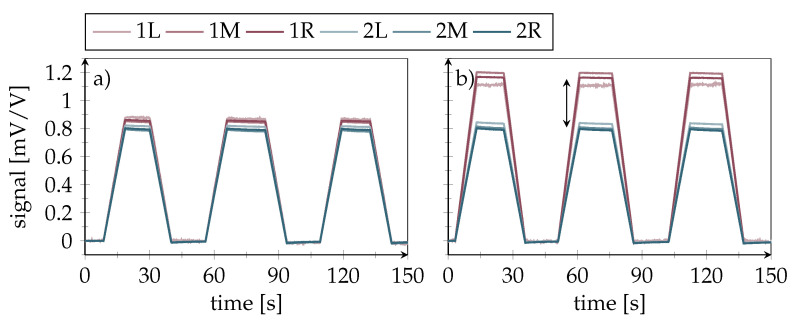
Sensor signals during cyclic quasi-static testing. (**a**) Artificial crack length of 16 mm; sensor signals between rows begin to deviate under load. (**b**) Artificial crack length of 23 mm; with increasing crack length, the signal amplitude of first row sensors rises. The colors indicate the first (red) and second (blue) sensor rows.

Figure 10b shows the averaged amplitudes of the sensors in rows 1 and 2, with each bar merging the signals from all three sensors in a row. While no signal difference could be observed at a crack length of 10 mm, a significant difference of up to 0.4mV$V^{-1}$ was seen for longer cracks, where the crack front distance to the inlay was 9 mm (crack length = 16 mm) and 2 mm (crack length = 23 mm), respectively. This shows that the differential signal rises before direct crack front contact. Moreover, the differential signal height can provide an estimate of the crack length. Figure 11 presents the output signals of the individual sensors inside the smart inlay over time during cyclic quasi-static loading. The sensors show good linearity and repeatability, although minor drift in the signals can be detected. A progressive signal difference with increasing crack length clearly proves the desired crack detection principle. However, once bondline damage has occurred, the differential signal becomes load-dependent. This can be seen in Figure 11b, where the slope of sensor row 1 exceeds that of row 2, which means that higher loads result in higher differential signals.

### 4.3. Fatigue Testing of Passive Bonds

**Figure 12:**
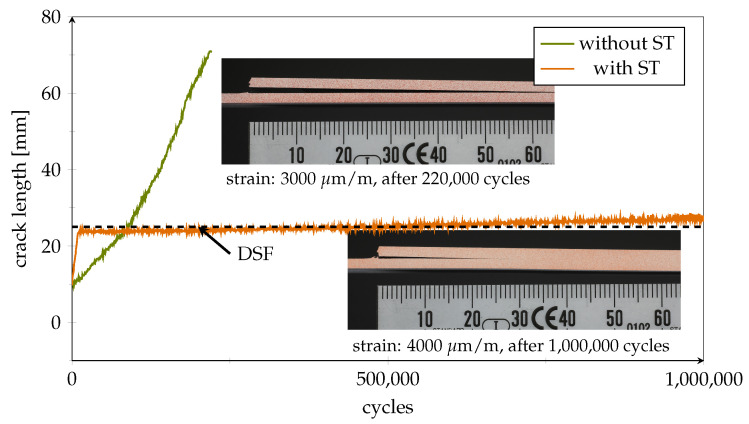
Comparison of optically determined crack progression during fatigue testing. Side views of a reference specimen without inlays (maximum strap strain: 3000 μm $m^{-1}$) and a sensorless specimen equipped with ST inlay (maximum strap strain: 4000 μm $m^{-1}$).

To simulate fatigue-induced continuous crack growth, healthy specimens were subjected to dynamic cyclic loading. Figure 12 exemplifies the difference between specimens with and without a crack arresting inlay (here without sensor structures). In the reference specimen without ST, a crack progressed quickly to a length of more than 65 mm (the end of our crack progression scale) within approximately 250,000 cycles using a maximum strain level of 3000 μm $m^{-1}$. In comparison, the specimen with ST showed some initial crack growth, but was still structurally intact when the fatigue test ended after one million cycles. Here, the crack remained almost stationary inside the first DSF at 25 mm even though the maximum load was set to 4000 μm $m^{-1}$.

### 4.4. Detection of Emerging and Progressing Cracks using Smart Inlays

**Figure 13:**
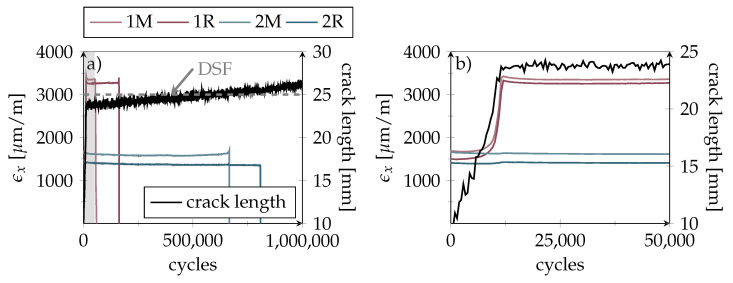
Fatigue testing results (maximum strap strain: 3500 μm $m^{-1}$) showing maximum strain values over cycles as measured by the smart inlay sensors. Colors indicate first (red) and second sensor row (blue). Optically measured crack length is depicted black while gray dotted line marks the DSF edge (**a**) Crack advances quickly to the first DSF where it becomes arrested. First sensor row gets destroyed early while second row sensors remain functional almost till the cycle ends. Area of first 50,000 cycles is marked with grey background. (**b**) Zoom of the first 50,000 cycles of the left plot. Difference between first and second sensor row signals clearly correlates with the crack length.

In the next step, the smart inlays were tested for their dynamic load-bearing capacity. All data shown in the following are from the same specimen, with the lap/strap geometry as shown in Figure 5. As Figure 13a shows, the crack was successfully stopped inside the first DSF, where it continued to propagate at a much lower pace; while, sensor signals provided plausible results in long-term load tests. This decisive progress compared to our earlier work on the smart inlay [20] was achieved by the addition of PEI-reinforcement for the sensing structures and the laser processes (Figure 1e–g). The second row sensors even stayed functional up to 700,000 cycles. As the zoomed plot in Figure 13b reveals, the first-row sensor signals correlate with increasing crack length, as expected. Once the crack was arrested in front of the inlay, the level of the measured first row strain indicates the applied load as expected and schematically illustrated in Figure 5.

**Figure 14:**
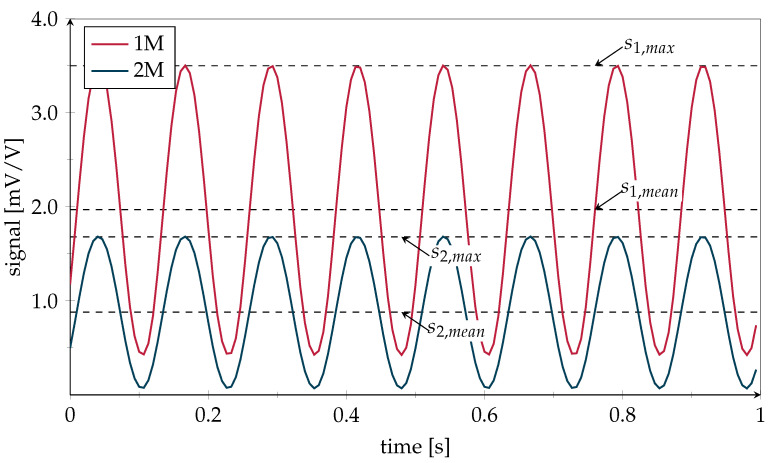
Exemplary sensor signal snapshot starting at 50,400 cycles when crack has reached the DSF. While the second row maximum value $s_{2,max}$ as well as the mean value $s_{2,mean}$ of sensor 2M remained at their initial values, the first row maximum amplitude $s_{1,max}$ as well as the mean value $s_{1,max}$ of sensor 1M increased with crack propagation.

Figure 14 shows a one second signal excerpt from the middle sensors at the point when the crack has reached the DSF. The signal oscillation corresponds to the applied load.

**Figure 15:**
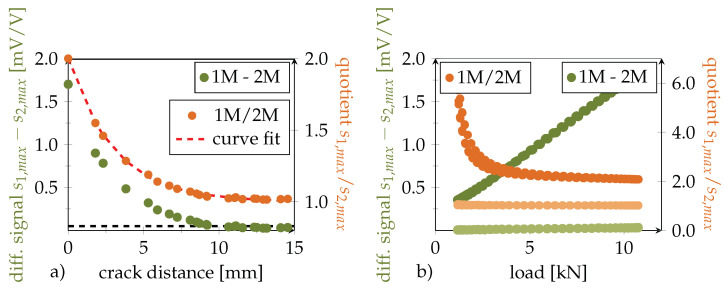
(**a**) Green curve corresponds to signal difference $s_{1,max}-s_{2,max}$ in dependence of crack distance. The black dashed line on the bottom represents a crack detection threshold level of 0.05 mV/V. The orange curve corresponds to $s_{1,max}/s_{2,max}$ and its fit forms the basis of the crack distance estimation algorithm. (**b**) Signal difference $s_{1,max}-s_{2,max}$ and signal ratio $s_{1,max}/s_{2,max}$ in dependence of load. Light green markers show the load independent initial differential relation (crack dist. = 15 mm), darker green markers the linear relation after 50,400 cycles (crack dist. = 0 mm). Same color wise allocation regarding crack distance was used for the orange quotient relation markers.

In order to display the following data independent of the selected inlay position within the bondline, the remaining *crack distance* to the first DSF is used in the following as a measure for crack propagation instead of total crack length. A simple threshold criterion for crack detection based on the differential signal is exemplified in Figure 15a by a horizontal black dashed line.

Depending on the required safety against measurement outliers and signal noise, this threshold must be adequately selected. However, as described earlier, the differential signal is not load-independent when a crack has occurred. This becomes also apparent in Figure 15b. Therefore, this criterion can only be used to generally recognize, not to quantify, bondline damage. With the exemplified threshold, the crack emergence signal is triggered approximately 10 mm before reaching the DSF, although only under the condition that the structure is fully loaded (here, 3500 μm $m^{-1}$).

The presented sensor design was developed to safely detect a crack when it has reached the DSF at the latest. However, as sensor signals rise upon crack emergence prior to DSF arrival, it seems feasible to find a signal-driven, load-independent estimation of the remaining crack distance *z* in front of the first DSF. The CFRP material is loaded only in its elastic regime. Thus, when the bond is loaded by an external load $F_{load}$, the strain sensor signals $s_{1}$ and $s_{2}$ can be expressed by

(2) $$\begin{array}{c} \underset{\frac{s_{1}}{s_{2}}=\frac{A_{1}\left(z\right)}{A_{2}}}{\underset{︸}{\to s_{1}=\frac{1}{E\cdot A_{1}\left(z\right)}\cdot F_{load}\to s_{2}=\frac{1}{E\cdot A_{2}}\cdot F_{load}}} \end{array}$$

In Formula (2), *E* is the Young’s modulus of the CFRP material and $A_{1}\left(z\right)$ and $A_{2}$ represent the effectively loaded CFRP cross-sections at the two measuring positions. As long as the bond is intact or the crack is far away from the smart inlay, $A_{1}\left(z\right)$ and $A_{2}$ are equal for both sensor rows. However, when a crack comes into the smart inlay proximity, the effective cross-section $A_{1}\left(z\right)$ decreases due to the lower load transfer into the lap. As the crack advances further, $A_{1}\left(z\right)$ progressively reduces depending on the thickness relation between the lap and the total thickness of the lap and strap. For our specimens, both adherends had a similar thickness; hence, $A_{1}\left(z\right)$ eventually reduced (when reaching the DSF) to half its initial value $A_{2}/2$, as the load is then carried by the strap cross-section only. By rearranging and inserting the similar components $F_{load}/E$ in Formula (2) into each other, it can be seen that the cross-sectional ratio equals the sensor signal quotient. Consequently the course of the signal quotient depends only on the effective cross-sections and is independent of the load. Assuming that the crack distance-dependant decay $A_{1}\left(z\right)/A_{2}$ can be described by an exponentially decreasing function, the sensor signal quotient $s_{1}/s_{2}$ can be expressed as

(3) $$\begin{array}{c} \rho \left(z\right)=e^{-z/a}+b=\frac{s_{1}}{s_{2}} \end{array}$$

To retrieve the analytical correlation, the experimental signal quotient ($s_{1,max}/s_{2,max}$) was fitted with this Formula, yielding a= 3.035 mm. Signal quotient and fit are plotted over the crack distance in Figure 15a. The value of *b* was approximated with the initial cross-sectional ratio $b=A_{1}\left(\infty \right)/A_{2}=1.0$, as the effective cross-sections are equal when the crack distance is large.

It should be noted that the fit value *a* provides an indication of the sensors’ detection range. The load transfer into the lap reaches 95% of its stable widespread level within a range of 3/β measured from the beginning of the overlap (this corresponds to the crack front) [1], where β=1/a using our notation. This yields a detection range of approximately 9 mm, which is the crack distance from the inlay at which detection is possible at the earliest. This seems to be in accordance with Figure 11, which showed for the static testing results a small but significant signal difference at a crack distance of 9 mm, corresponding to a crack length of 16 mm.

In contrast to the differential criterion described earlier, the quotient relation remains stable for higher loads, as shown in Figure 15b. However, for smaller loads the quotient is sensitive to small but stable signal offsets between a sensor pair appearing when the joining partners initially settle under load. This means that a quotient criterion can be used to estimate crack distance independent of load once a certain minimal level of loading can be assumed (here, approximately 4 kN). To further improve this, suitable pre-calibration steps which eliminate any offset between the sensor pairs in the loaded healthy bondline state can be conducted.

The crack distance estimation via signal quotient using the fit value *a* can be expressed as

(4) $$\begin{array}{c} \Leftrightarrow z=-a\cdot \ln(s_{1}/s_{2}-1) \end{array}$$

**Figure 16:**
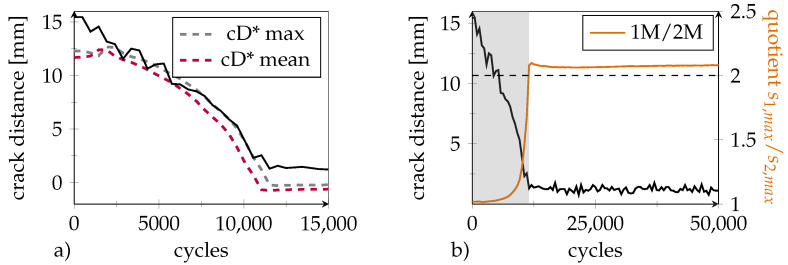
(**a**) Optically measured remaining crack distance from the DSF (black) and crack distance (cD*) estimated from the signal ratios of sensor 1M and 2M at two different load levels ($s_{max}$ and $s_{mean}$). (**b**) Course of $s_{1,max}/s_{2,max}$ (orange) with progressing crack. Grey area marks region of continuous crack progression. Initially the ratio assumes a value of 1 but increases with crack propagation. As soon as the crack reaches the first DSF, the ratio assumes a value of 2 and optical evaluation (black) reveals that the crack has stopped.

As shown in Figure 16a, crack distance estimation based on the signal quotient is in good correlation with the measured length for two load levels. In addition to the maximum values, $s_{1,max}$ and $s_{2,max}$, the mean values $s_{1,mean}$ and $s_{2,mean}$ of the signals during cyclic loading were used. This illustrates that the estimating calculation successfully suppresses the influence of load. However, deviations remain for the time of crack emergence (crack distance 15 mm) as well as for the zero value immediately in front of the DSF. Regarding the former, this is because the slope of the correlation between the crack distance and signal quotient is rather flat in this area, which limits the detection range (refer to Figure 15a). The latter is likely to be caused by measuring inaccuracies of the actual crack distance, as the optical sideview image evaluation is subjected to a certain non avoidable degree of uncertainty.

From Figure 15a, it can be seen that the effective cross-sectional ratio at a crack distance of zero equals $A_{1}\left(0\right)/A_{2}=s_{1}/s_{2}=2.0$. This level is marked as a dashed line in Figure 16b. As for the other ST equipped specimens, the crack propagated almost linearly towards the DSF within the first approximately 10,000 cycles. The intersection point with the dashed threshold marks the moment when the crack reached the first DSF. This observation can be exploited to define a binary zero crack distance criterion that indicates an urgent need for repair.

## 5. Conclusions and Outlook

The results gained from the mechanical testing of smart inlay-equipped specimens have shown that a full functionally compliant compliant implementation is possible. Based on FE simulations that revealed a confined but highly strained zone in the vicinity of a stress peak, which usually occurs in front of the first DSF, sensor placement was adjusted to avoid damage due to overloading. In addition, DSF simulations in both the in-plane and through-thickness directions revealed the positive influence of PEI-reinforcement in combination with a protective PVDF cover layer. This reduces shear deformation at the sensor location without influencing the longitudinal in-plane strain that needs to be measured. Furthermore, the additional layer improves handling robustness upon integration. The new PEI-reinforced inlay proved its crack detection capabilities in a test setup under static loading and with different lengths of artificial cracks. With the dynamic fatigue tests, a more realistic scenario with stress-related crack propagation was created. The results show that PEI-layer-reinforced sensors are on the verge of completely solving any durability issues. From the first row sensor data, it can be seen that these sensors stayed functional considerably longer time (up to 200,000 cycles) than the point when the crack has reaches the DSF (within approximately 10,000 cycles). The best second row sensor stayed fully functional for as many as 800,000 cycles. Moreover, the system showed promising results regarding crack detection within the first 50,000 cycles, as well as advanced capabilities such as a detection of the point of time when the crack has reached the DSF and crack distance estimation solely based on the quotient between the signals of both rows. This estimation is independent of the actual load condition, and therefore perfectly suited for real situations, e.g. in aircrafts, where the momentary load is highly variable and unknown and confidence about structural integrity valuable.

For future samples, an alternative electrical contacting concept should be considered, as the lateral track bridge experiences high mechanical stress. Likewise, the presented crack length estimate should be comprehensively validated in order to check the general validity of the fitted parameters in practice. Finally, even though the presented system has proven functional, the future focus of development can aim for a more cost-effective inlay manufacturing processes such as screen printing. Only then industrial applicability can be achieved.

## Figures and Tables

**Table 1:** Overview of periodic load levels for 1,008,000 cycles.

Max. Strain	Load level_mean_ ± Oszi. Amplitude	F_max_
3000 μm $m^{-1}$	5.104 kN ± 4.176 kN @ 8 Hz	9.280 kN
3500 μm $m^{-1}$	5.973 kN ± 4.887 kN @ 8 Hz	10.860 kN
4000 μm $m^{-1}$	6.837 kN ± 5.594 kN @ 8 Hz	12.431 kN

## Abbreviations

The following abbreviations are used in this manuscript:

CFRP	carbon fiber-reinforced plastic
CLS	cracked lap shear
DSF	disbond-stopping feature
FE	finite element
MDAF	multifunctional disbond arrest feature
PEI	polyetherimide
PVDF	poly(vinyliden fluoride)
ST	surface toughening
